# Role of Inflammation and Oxidative Stress Mediators in Gliomas

**DOI:** 10.3390/cancers2020693

**Published:** 2010-04-26

**Authors:** Alfredo Conti, Carlo Gulì, Domenico La Torre, Chiara Tomasello, Filippo F. Angileri, M’Hammed Aguennouz

**Affiliations:** Department of Neuroscience and Department of Oncology, University of Messina, Policlinico Universitario, Via Consolare Valeria 1, 98125, Messina, Italy

**Keywords:** inflammation, oxidative stress, nuclear factor-kappaB, glioma, brain tumor

## Abstract

Gliomas are the most common primary brain tumors of the central nervous system. Despite relevant progress in conventional treatments, the prognosis of such tumors remains almost invariably dismal. The genesis of gliomas is a complex, multistep process that includes cellular neoplastic transformation, resistance to apoptosis, loss of control of the cell cycle, angiogenesis, and the acquisition of invasive properties. Among a number of different biomolecular events, the existence of molecular connections between inflammation and oxidative stress pathways and the development of this cancer has been demonstrated. In particular, the tumor microenvironment, which is largely orchestrated by inflammatory molecules, is an indispensable participant in the neoplastic process, promoting proliferation, survival and migration of such tumors. Proinflammatory cytokines, such as tumor necrosis factor-alpha, interleukin-1beta, and interferon-gamma, as well as chemokines and prostaglandins, are synthesized by resident brain cells and lymphocytes invading the affected brain tissue. Key mediators of cancer progression include nuclear factor-kappaB, reactive oxygen and nitrogen species, and specific microRNAs. The collective activity of these mediators is largely responsible for a pro-tumorigenic response through changes in cell proliferation, cell death, cellular senescence, DNA mutation rates, DNA methylation and angiogenesis. We provide a general overview of the connection between specific inflammation and oxidative stress pathway molecules and gliomas. The elucidation of specific effects and interactions of these factors may provide the opportunity for the identification of new target molecules leading to improved diagnosis and treatment.

## 1. Introduction

Malignant gliomas are the most common primary brain tumors. Adult patients diagnosed with a malignant glioma almost invariably face a dismal prognosis with a life-expectancy of less than 12 months from diagnosis [1]. Major advancements in the knowledge of the biology of these tumors have been achieved, and recent years have witnessed remarkable advances in neuroimaging, surgical techniques and adjuvant therapies. Unfortunately, the translation of current knowledge in clinical practice still yields disappointing results, with local recurrence being the most common modality of relapse.

The development of malignant gliomas is a multistep process, during which genetic alterations confer specific types of growth advantages [2]. Malignant growth is characterized by several key changes: self-sufficiency of growth signals, insensitivity to antigrowth signals, escape from apoptosis, unregulated proliferation, enhanced angiogenesis, and acquisition of invasive properties [2]. Each of these shifts is complicated and accomplished by combined efforts of various signaling processes. Among a number of different biochemical events, the existence of molecular connections between inflammation and oxidative stress pathways and the development of this cancer has been demonstrated. A well-regulated inflammatory response can be anti-tumorigenic and have a role in tumor suppression [3]. On the other hand, chronic inflammation is detrimental and, among other deleterious effects, frequently predisposes cells for an oncogenic transformation. Both extrinsic and intrinsic inflammation pathways may be carcinogenic [4]. In the extrinsic pathway, chronic inflammation and/or infection is the driving force that causes the increase in cancer risk [5,6,7,8]. Alternatively, in the intrinsic pathway, genetic alterations of oncogenes and/or tumor suppressor genes are the primary cause of cancer. These genetic alterations affect the expression of various inflammatory genes and leads to recruitment of inflammatory cells. This is the reason why nearly all tumors have inflammatory cells present in the tumor or tumor microenvironment regardless of the underlying cause of the tumor. Common inflammatory mediators including cytokines, chemokines, reactive oxygen and nitrogen species (RONS), cyclooxygenase-2 (COX-2) and nuclear factor (NF)-κB can lead to cellular conditions favorable for tumor promotion. This release of inflammatory molecules can also cause reduction of cell-mediated cytotoxicity and potential immune evasion for tumors. In glioma development, the tumor microenvironment, which is largely orchestrated by inflammatory molecules, is an indispensable participant in the neoplastic process, fostering proliferation, survival and migration of such tumors.

In this review, we provide a general overview of the connection between specific inflammation and oxidative stress pathway molecules, microRNAs and gliomas. The elucidation of specific effects and interactions of these factors may provide the opportunity to identify new target molecules, leading to improved diagnosis and treatment.

## 2. Cytokines Escape from the Immune Response in Gliomas

The biological “success” of gliomas proves that immunologic mechanisms do not function properly within these tumors. In fact, glioma cells can locally impair the immune response and cytokines play a pivotal role in this immunosuppressive process. 

Rossi *et al.* [9,10] pointed out how a surprisingly high number of macrophages can be retrieved within gliomas. In fact, while T lymphocytes are rather rare in gliomas, approximately one-third of all cells in glioma biopsies are labeled by macrophage markers. Those can be intrinsic cells of the central nervous system (CNS) and blood-borne monocytes/macrophages [11,12]. Graeber *et al.* in 1994 [13] identified a large number of MHC class II-positive microglial cells intimately associated with tumor cells. This association suggested that the main immune response against glioma is related to the antigen presentation operated by the microglia and the consequent activation of cytotoxicity by T cells patrolling the CNS. This is a multistep process involving the presentation of tumor antigens by an antigen presenting cell expressing a MHC class II molecule. A co-stimulating activity is provided by other cell surface molecules, such as B7-1 and soluble factors, such as the cytokine interleukin-1 (IL-1). CD4+ T lymphocytes (T helper) subsequently activate cytotoxic CD8+ T cells that recognize tumor antigen in the molecular context of MHC class I antigen.

In recent years, it has become increasingly clear that the immune defense functions of microglial cells against glioma are compromised and that deficient antigen presentation may be an underlying cause [14]. In order to activate the cytotoxicity, the CD4+ T cell produces a number of cytokines to activate the CD8+ T lymphocyte and amplify the response. Those cytokines include IL-2, IL-4, IL-7, IL-12 and INF-γ. Once activated, the cytotoxic T cell is programmed to kill cells bearing the antigen, associated with a MHC class I, which induced its differentiation and activation. Two mechanisms may follow the activation of the cytotoxic T lymphocytes: the production of a protein able to polymerize and create ion channels in the cell membrane, named “perforin”, with a subsequent osmotic lysis; the activation of apoptotic mechanisms through a FAS/TRAIL receptor-associated pathway.

Nevertheless, this above described mechanism can be suppressed by immuno-inhibitory products synthesized by glioma cells. The immunosuppressive factors include the transforming growth factor (TGF)-β, the IL-10, the insulin-like growth factor I, and the prostaglandin E2 (PGE2).

The TGF-β is a potent regulator of the growth and functions of lymphocytes and macrophages [15]. Human GBM cell lines have been shown to produce the isoform TGF-β2. This appears to be involved in the regulation of both suppression of anti-tumor immune surveillance and angiogenesis in malignant gliomas [16]. 

A second soluble factor produced by malignant gliomas and involved in the immunosuppressive properties of such tumors is the PGE2. The PGE2 activates a different family of receptors causing the downregulation of the activity of the lymphokines-activated killer (LAK) cells. PGE2 can also suppress the proliferation of T-cells induced by mitogen antibodies anti-CD3 *in vitro* [17]. The PGE2 also causes the downregulation of the expression of the MHC class II HLA-DR, possibly contributing to the ability of the tumor cells to escape the immune surveillance.

A third factor produced by malignant gliomas and involved in the local immunosuppression is the cytokine IL-10. Levels of IL-10 expression are higher in malignant gliomas with 87.5% of grade III and IV and only 4% of grade II tumors showing high levels of IL-10 mRNA [18]. Indirect studies demonstrated that IL-10 inhibits the release of the INF-γ from the T helper lymphocytes and partially inhibits the expression of the MHC class II molecules on macrophages/microglial cells [19].

## 3. The Link between Inflammation and Cancer: The Nuclear Factor-κB

Nuclear factor-κB is a generic term to identify a family of dimeric transcription factors. The NF-κB is a key regulator of inflammatory gene expression [20,21,22,23] that has been shown to activate, via transcription, genes encoding proinflammatory cytokines (TNFα, IL-lβ, and IL-12), cell adhesion molecules (vascular cell adhesion molecule-1 and intercellular cell adhesion molecule-1), iNOS, and COX-2. These molecules, together with NO derived from iNOS and COX-2-produced PGE2, play important roles in the pathogenesis of inflammation and neurodegenerative disease, but also of cancer genesis and progression.

A linkage between NF-κB and cancer was supposed as early as the association between c-Rel and its viral oncogenic derivative v-Rel was recognized [24]. Over the past few years, constitutive NF-κB activation has been described in a variety of epithelial and lymphoid cancers. In general, as no loss-of-function mutations of the inhibitory subunit IκB or gain-of-function IκB kinase mutations have been detected, it has been suggested that NF-κB activation in cancer may be the result of either exposure to proinflammatory stimuli in the tumor microenvironment or mutational activation of upstream components in IκK - NF-κB signaling pathways [25].

**Figure 1:**
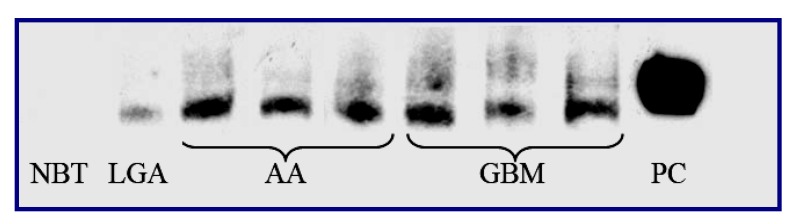
NF-κB DNA-binding activity studied in normal brain tissue and human gliomas with different grade of malignancy. Representative electromobility shift assay autoradiographs showing NF-κB DNA-binding activity studied in normal brain tissue and human gliomas with different grade of malignancy and demonstrating an increased activity in tumor samples that is remarkable in high grade tumors. NBT: normal brain tissue; LGA: low-grade glioma; AA: anaplastic astrocytoma; GBM: glioblastoma multiform; PC: positive control.

Concerning gliomas, Nagai *et al.* [26] and Gill *et al.* [27] provided evidence for a role of this transcription factor in the proliferation and survival of glioblastoma cell lines. Weaver *et al.* [28] also later reported that NF-κB activation in response to chemotherapeutic agents somewhat protected U87 and U251 glioblastoma cells *in vitro*. We recently reported the presence of aberrant NF-κB DNA-binding activity in WHO Grades II to IV astrocytomas surgically removed from humans compared with normal brain tissue [29]. Our data also showed a trend toward the correlation of nuclear activity with tumor grade. The increased NF-κB-related transcriptional activity indicated that glioma cells exist in an activated state and that the degree of such abnormal activation increases progressively with malignancy [29] (Figure 1).

The main role of NF-κB in the cell immortalization process seems to be related to the control of apoptosis. Actually, NF-κB protects fibroblasts against TNFα-induced apoptosis, as demonstrated with cells from RelA-deficient mice [30]. Furthermore, Jurkat or fibroblastic cells undergo enhanced apoptosis after treatment with TNFα, ionizing radiation, or daunorubicin when nuclear translocation of NF-κB is blocked [31,32,33]. The mechanism by which apoptosis is blocked by the nuclear translocation of NF-κB is related to the expression of a series of antiapoptotic genes. Based on their NF-κB-dependent expression and antiapoptotic function, genes encoding for c-IAP1, c-IAP2 (both inhibitors of apoptosis protein), TRAF1, A20, BCL-2, BCL_XL_, survivin and XIAP have been proposed as target genes in such a mechanism.

**Figure 2:**
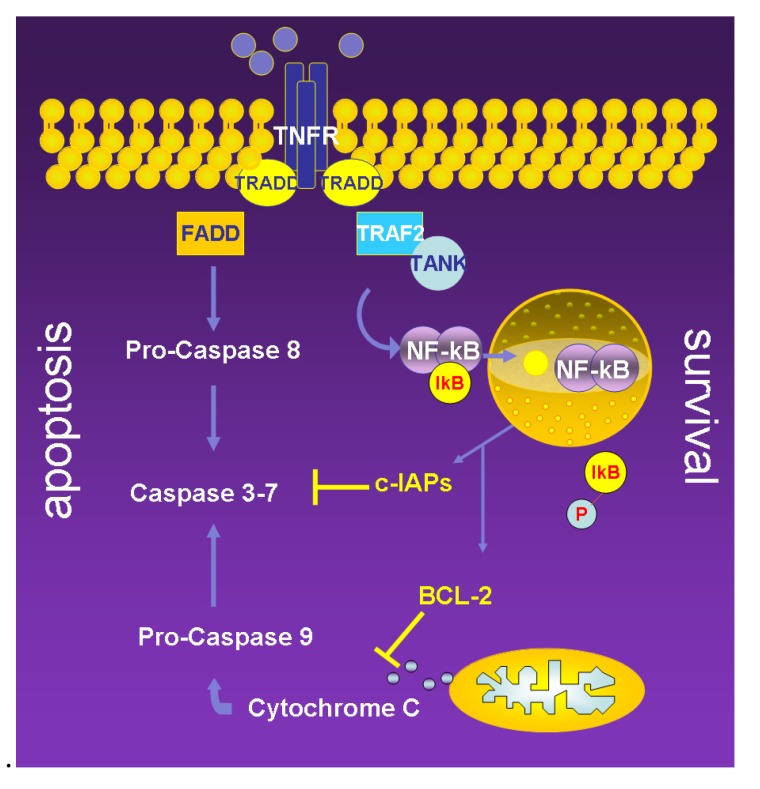
Schematic drawing showing the TNF-driven survival and apoptosis balance. TNF signaling induces controlled cell death through the caspase activation cascade. This is counteracted by a survival pathway acting through a TRAF2-driven and NF-kB-mediated transcription of antiapoptotic proteins including members of the c-IAP family (c-IAP1 and 2 and Survivin) acting on the effector caspases 3 and 7 or members of the Bcl-2 family acting on the mitochondrial apoptotic pathway by blocking the release of cytochrome c. TNFR = tumor necrosis factor receptor; TRADD = TNFR associated death domain; FADD = FAS associating protein with death domain.

The signaling cascade that leads to the activation and antiapoptotic activity of NF-κB is mediated by TNF stimulation, as shown by the experimental evidence that TNF or soluble CD40L induces nuclear translocation of NF-κB [34]. The signal transduction mechanism emanating from the TNF-receptor is thought to be mediated by the TNF-receptor associated factor (TRAF) 2, a signaling intermediate that has been shown to be recruited to the cytoplasmic tail of the TNF-receptor through a TNFR1/TRADD/TRAF2 interaction. Based on this hypothesis, TNF can either induce apoptosis through FADD (FAS associating protein with death domain) or TRADD (TNF-receptor associated protein with death domain) and caspase recruitment or promote survival through TRAF2 recruitment and NF-κB and caspase recruitment or promote survival through TRAF2 recruitment and NF-κB induction (Figure 2) [30,31,35]. 

Recently we demonstrated that antiapoptotic proteins such as Bcl-2, TRAF1, and members of the IAP family of genes, particularly survivin, can be found in human astrocytic tumors when compared with normal brain tissue. This overexpression is associated with the aberrant transcriptional activity of NF-κB recruited through TNF/TNF-receptor/TRAF2 activation. Further, we demonstrated that the expression of antiapoptotic factors, particularly those of Bcl-2 and survivin, changed significantly in relation to tumor grading, with higher expression levels of BCL-2 in low grade tumors and strongly prominent expression of survivin in high grade tumors [36].

Accordingly NF-κB may links TNFα and glioma progression, but its role in cancer seems to be more complex [25]. For instance, NF-κB might also be involved in linking inflammation to cancer by induction of other proinflammatory cytokines, such as IL-6 and, and chemokines, such as IL-8, adhesion molecules, MMPs, COX-2, and iNOS.

In an experimental model for colitis-associated cancer it has been demonstrated that there is an activation of NF-κB in macrophages of the lamina propria. This results in the production of cytokines, particularly IL-6, which drive the proliferation of premalignant intestinal epithelial cells. IL-6 then exerts its proliferative effect on the intestinal epithelial cells, through the activation of another transcription factor, the signal transducer and activators of transcription 3 (STAT3), which further synergizes with NF-κB to increase the expression of survival genes [25]. 

**Figure 3:**
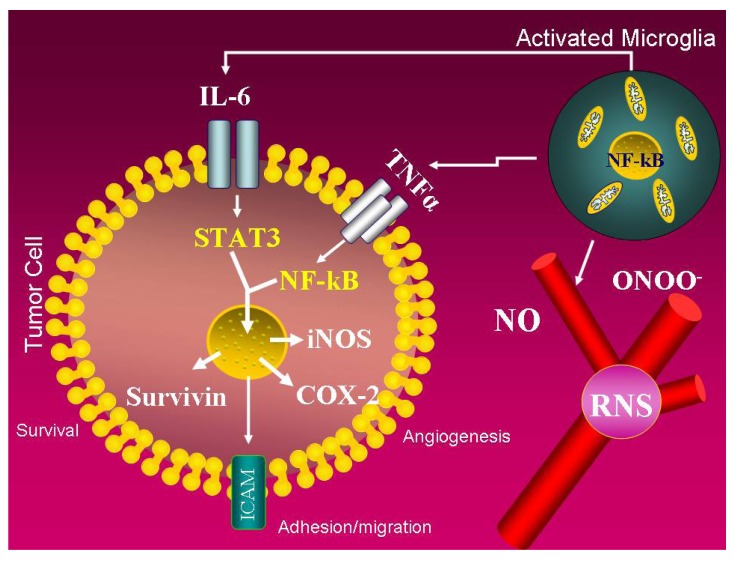
Possible IL-6-mediated paracrine activation of glioma cells. Schematic drawing describing the hypothesis that inflammatory cells, such as activated macrophage/microglial cells, synthesize IL-6 through NF-κB. As a consequence, glioma cells, particularly tumor stem cells, are activated by IL-6 via paracrine stimulation. Chemokines, including IL-6 and TNFα, activate the transcription factors STAT3 and NF-κB that, in turn, initiate specific pathways for tumor progression such as angiogenesis, migration, and apoptosis inhibition.

**Figure 4:**
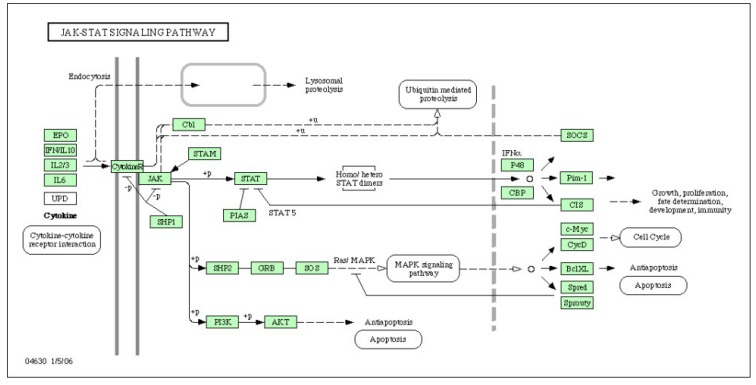
Cytokines-related activation of intracellular processes in glioma cells. Glioma specimens were hybridized in a GeneChip microarrays panel of the whole human genome containing approximately 44000 genes. The scheme presented results from statistical analysis as displayed by Gene Spring GX 7.3 software (personal, unpublished data).

These results suggest that some of the protumorigenic effects of NF-κB activation in myeloid cells could be caused by paracrine signaling to STAT3 in epithelial cells (Figure 3). Although no similar studies have been conducted in glioma models, it has been recently demonstrated that IL-6 is clinically significant, because elevated IL-6 ligand and receptor expression are associated with poor glioma patient survival. Furthermore, glioma stem cells (GSCs) preferentially express two IL-6 receptors: IL-6 receptor alpha (IL-6Rα) and glycoprotein 130 (gp130). Targeting IL-6Rα or IL-6 ligand expression in GSCs with the use of short hairpin RNAs (shRNAs) significantly reduces growth and neurosphere formation capacity while increasing apoptosis. Perturbation of IL-6 signaling in GSCs attenuates signal transducers and STAT3 activation, and small molecule inhibitors of STAT3 potently induce GSC apoptosis. These data indicate that STAT3 is a downstream mediator of prosurvival IL-6 signals in GSCs [37] (Figure 4). The hypothesis that activated macrophage/microglial cells may produce IL-6 through NF-κB and that glioma cells, particularly tumor stem cells, may be activated by IL-6 through a paracrine mechanism deserve further studies.

NF-κB is also involved in the control of production of the chemokine IL-8 in gliomas [38]. Interleukin-8 belongs to a specific group of chemokines called CXC, for the presence of two cysteines separated by a single amino acid in the peptide sequence. In inflammation IL-8 is secreted by activated monocytes and macrophages, and promotes the directional migration of neutrophils, basophils and T lymphocytes. The IL-8 gene promoter has specific binding sites for transcriptional modulators AP-1, NF-κB, C-EBP/NF-IL-6 and up-regulation occurs in the setting of synergistic interaction of transcriptional factors, especially during oxidative stress or hypoxia/anoxia stimulation. IL-8 exerts its function through linkage with three different kind of G-protein-coupled chemokines receptors CXCR1, CXCR2 and Duffy antigen receptor for cytokines (DARC). Both CXCR1 and CXCR2 are expressed by leucocytes and endothelial cells, and mediate important functions such as neutrophil recruitment and angiogenesis. CXCR2 is also present in CNS neurons. Specifically in glioma cells IL-8 is a potent mediator of angiogenesis and through at least four distinct pathways concerning stimulation of endothelial proliferation, inhibition of apoptosis in endothelial cells, increase of endothelial cell mRNA expression of matrix metalloproteinases (MMPs) and formation of capillary tube. All these cited activities depend on the endothelial cell expression of receptors CXCR1 and CXCR2, are independent of other pro-inflammatory effects, occur in a dose dependent manner and may be blocked by monoclonal antibodies to IL-8 [39,40]. In addition to angiogenic activity, recent investigations have reported that IL-8 plays a key role in gliomagenesis probably because it acts as inflammatory chemoattractant as part of the host response to neoplasia and it represents a more general pro-inflammatory autocrine and paracrine growth factor released in response to tissue stress and necrosis, promoting development of neoplasia.

## 4. Nitric Oxide and Its Controversial Role in Gliomagenesis

Reactive oxygen species and reactive nitrogen species (collectively RONS) are highly reactive radicals that contain unpaired valence shell electrons. They have an important role in the innate immune system. In response to a stimulus, phagocytic cells release RONS and non-phagocytic cells are stimulated to produce RONS by pro-inflammatory cytokines [41].

Nitric oxide (NO) is one type of reactive nitrogen species. Nitric oxide is synthesized from L-arginine by the NO synthase (NOS). Three isoforms of the NOS have been described. Two isoforms are expressed constitutively (cNOS), the neuronal (nNOS or type 1) and the endothelial (eNOS or type 3), and one inducible under pathological conditions (iNOS or type 2) [21]. Induction of iNOS requires inflammatory cytokines, such as IFNγ, TNFα, and IL1β. This induction occurs at the transcriptional level through the activation of transcription factors. While iNOS induction by IFNγ is mediated by activation through STAT1, TNFα and IL-1β acts via NF-κB [20,29,42,43]. The cytotoxic effects of NO are partly due to the production of peroxynitrite, a reactive oxidant formed by the rapid reaction of NO and superoxide [44,45]. 

The production of RONS by phagocytes induces cell death by phagocytic destruction and apoptosis, for instance oxidative stress induces p53 protein accumulation in glioma cells directing them to die by apoptosis [46]. On the other hand, increased RONS, creating increased oxidative and nitrosative stress, may contribute to carcinogenesis [41,47,48]. With regard to gliomas, iNOS expression and activity are upregulated in rodent experimental models such as the C6 [49]. Human gliomas have presented a complex picture. In fact, NO expression and NOS function are increased, and NOS expression correlated with the degree of malignancy [50,51]. Furthermore, in human gliomas, a redistribution of NOS isoform activity has been suggested. Garbossa *et al.* reported a reduced activity of nNOS in the peritumoral cortex, with a marked increase in iNOS immunoreactivity within reactive and tumor glial cells and the endothelium of small blood vessels in edematous regions [52]. Broholm *et al.* described an increase of nNOS expression in tumor cells that was more pronounced in high-grade tumors; in this study eNOS was sporadically expressed in tumor cells, but was increased in endothelial cells in both the tumor vasculature and peritumoral areas [53]. 

According to these data, NOS activity is upregulated in gliomas with a consequential oxidative stress. Increased peroxynitrite levels may lead to DNA strand breaks, point mutations and aberrant DNA cross-linking, thereby causing genomic instability. This contributes to carcinogenesis by mutating proto-oncogenes and tumor suppressor genes [54]. For instance, Cobbs *et al.* [54] demonstrated that peroxynitrite and SIN-1 (3-morpholinosydnonimine hydrochloride), a molecule that produces peroxynitrite by simultaneously generating NO and superoxide, can inhibit wild-type p53 protein transcriptional activity. Concentrations of peroxynitrite similar to those that occur during inflammatory conditions and malignancy can cause posttranslational modifications of wild-type p53 protein that are associated with dysregulation of p53 transcriptional activity and downstream pathways, particularly cell cycle checkpoint controls. It is therefore conceivable that with prolonged exposure of cells to increased concentrations of RONS, as might occur in a chronic inflammatory process or malignancy, oncogenic progression could develop. Other studies have demonstrated that constituents of the epidermal growth factor receptor, Ras, and p120 Src signaling pathways can become activated by peroxynitrite [55,56,57], and thus cell proliferation, growth arrest, and apoptotic pathways are likely affected by peroxynitrite through a complex interplay of multiple activating and inhibitory signaling pathways in glioma cells.

Also lipid peroxidation products, such as malondialdehyde and 4-hydroxynonenal, can form DNA adducts that can lead to point mutations [58]. These reactive molecules may also generate inflammatory stimuli to propagate the effect [59]. RONS can post-translationally modify various proteins, rendering them auto-antigenic (*i.e.,* inflammatory), and may also increase phosphorylation and inactivate the retinoblastoma 1 tumor suppressor protein and thus lead to cell proliferation [60]. Furthermore, elevated RONS can increase angiogenesis and transcriptional activation of oncogenes [61]. 

Noticeably, studies using a wide range of *in vitro* and *in vivo* models show that iNOS/NO signaling can also induce COX-2, which itself is a promising link between inflammation and cancer [62].

## 5. Cyclooxygenase in Glioma Biology

**Figure 5:**
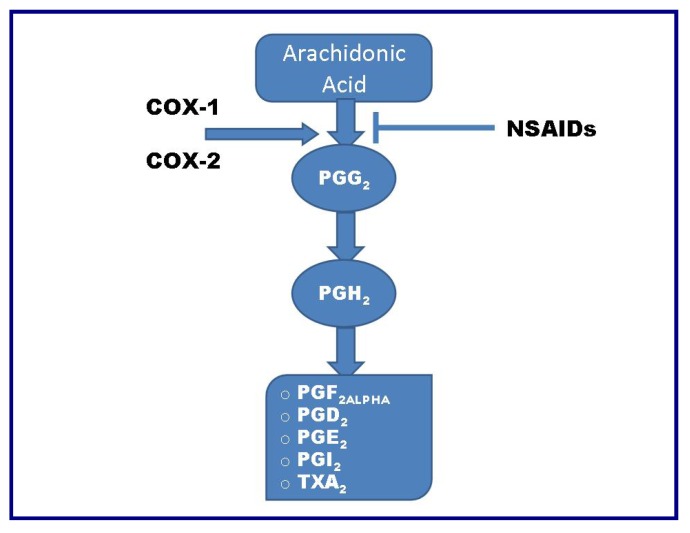
Schematic representation of the conversion of arachidonic acid to prostaglandins and other prostanoids.

Cyclooxygenase is a key enzyme involved in the transformation of arachidonic acid to important biological molecules that play a key role in inflammation. Such molecules include prostaglandins, prostacyclin and thromboxane. The target of COX is arachidonic acid, which is converted to prostaglandin H2 (PGH2), the precursor of the series-2 prostanoids (Figure 5). Three COX isoforms have been identified: COX-1, COX-2, and COX-3. COX-3 is a splice variant of COX-1, which retains intron one and has a frameshift mutation; thus the alias name of COX-1b or COX-1 variant (COX-1v) [63]. Both COX enzymes seem to carry out the same catalytic reaction. They have a similar molecular structure [64], but COX-1 is constitutively expressed in nearly all normal tissues and mediates the synthesis of prostaglandins required for physiological tissue homeostasis. In contrast, COX-2 expression is inducible and increases in response to various stimuli, including inflammatory signals, mitogens, cytokines, and growth factors.

Because COX is involved in several biological processes such as inflammation, angiogenesis, pain, platelet aggregation, a potential role in carcinogenesis has been supposed as well. The studies on COX and cancer show that pre-malignant lesions, early stages and late stages of cancer express increased COX-2 levels, suggesting that COX-2 has an important role in both tumor initiation and maintenance [65].

In different studies [66,67], high COX expression turned out to be relevant in gliomas and predictive of poor prognosis. Deininger *et al.*. detected COX-1 in 20-50% of all cells in both low- and high-grade gliomas. Of the COX-1-positive cells, 90% expressed MHC class II antigens, whereas no COX-1 immunoreactivity was observed in tumor cells [67]. 

Joki *et al.* found that the COX-2 protein was expressed in all human glioma specimens tested; moreover, the percentage of positively stained cells was significantly higher in high-grade gliomas than in low-grade gliomas or normal brain [68]. This correlation between the expression levels and the degree of glioma malignancy have been confirmed by Shono *et al.* who also reported that high COX-2 staining predicts a poor prognosis in patients with glioma in general and in patients with glioblastoma multiform (WHO grade IV) in particular [66].

Nonetheless, the exact role of COX in glioma biology remains unclear. It has been reported that increasing COX-2 activity may reduce the apoptotic tendency of intestinal epithelial cells in rats. Moreover, forced expression of exogenous COX-2 in colon cancer cells increased their metastatic potential and invasiveness, and COX-2 stimulates the release of proangiogenic prostaglandins, promoting endothelial cell migration and tube formation, which are the initial steps in angiogenesis [69]. 

Although each of these mechanisms has not been specifically studied in gliomas, Joki *et al.* demonstrated that NS-398, a COX-2-specific inhibitor, increased apoptosis, reduced proliferation, and attenuated invasion of cultured human glioma cells [68]. Consequently, COX-2, through prostaglandins and other actions, may influence many of the aggressive features of gliomas. 

In 2007, Kang *et al.* [70] demonstrated that the selective COX-2 inhibitor celecoxib enhances glioblastoma sensitivity to ionizing radiation. The authors analyzed proliferative and angiogenic rates analyzing angiopoietin-1, angiopoietin-2, and vascular endothelial growth factor (VEGF). Celecoxib enhanced U-87MG cell radiosensitivity. Angiopoietin-1 and VEGF proteins decreased, whereas angiopoietin-2 expression increased. Furthermore, *in vivo*, median survival of control mice intracranially implanted with U-87MG cells was 18 days, whereas celecoxib significantly extended median survival of irradiated mice to 41 days, with extensive tumor necrosis compared with irradiation alone. 

Bijnsdorp *et al.* used a panel of three glioma cells lines (D384, U87 and U251) to investigate the effects of the selective COX-2 inhibitor meloxicam alone and in combination with irradiation *in vitro*. The exposure to 250-750 micromolar meloxicam resulted in a time- and dose-dependent growth inhibition with an almost complete inhibition after 24 h for all cell lines [71]. An interesting finding in this topic is the relationship between COX and angiogenesis, and its possible therapeutic implication. 

As reported by Masferrer *et al.*, COX-2 activity seems to be strongly associated with tumor angiogenesis. The Authors demonstrated a relevant antitumor effect of celecoxib in tumor models in which only the tumor endothelial cells were expressing COX-2 [72].

Recent studies on COX-2 inhibitors also suggests a mechanism involving the suppression of neovascularization [73]. Wagemakers *et al.* implanted syngeneic GL261 glioma cells in C57bl/6 experimental mice. Mice were i.p. injected with the COX-2 inhibitor E-6087 and its metabolite E-6132 [73]. The mean tumor volume of the group treated with the combination of COX-2 inhibitor and fractionated radiation was the lowest of all treatment groups, and in particular, this volume was significantly smaller than the mean tumor volume of the group treated with radiation only. For what concerns angiogenesis and tumor growth, the Authors demonstrated that inhibition of COX-2 caused “vessel normalization” namely a process that counteracts changes in vascular behavior induced by the angiogenic process. Accordingly, it is hypothesized that tumor angiogenesis can be initiated only after a switch in the balance of angiopoietin-1 and angiopoietin-2 in favor of the latter. COX-2 inhibition may lead to vessel normalization possibly blocking the ability of the tumor to induce an effective angiogenic switch and thereby inhibiting tumor outgrowth, even though further studies needs to be conducted to demonstrate the biochemical mechanism of these phenomena.

Recently it has been reported that one of the most important causes of glioma resistance to therapy is related to cancer stem cells [74]. Glioma stem cells form a small subpopulation of cells within the brain tumor responsible for the initiation and maintenance of the tumor mass [75]. The major feature of CSCs is the ability to form neurospheres, which is free-floating structure generated *in vitro*. When cultured in neurosphere conditions, CSCs express a very high level of the neural precursor cell marker CD 133 (Prominin-1). CD133 is associated with high levels of membrane-type matrix metalloproteinases (MT-MMP) and COX-2 [74]. A molecular signaling convergence linking COX-2 to MT1-MMP expression in glioma cells, and in particular in CSCs, possibly acting on tumoral neoangiogenesis trough a mechanism PGE2-induced deserves particular attention.

## 6. Molecular Epidemiology to Identify Inflammation-Related Risk Factors

**Figure 6:**
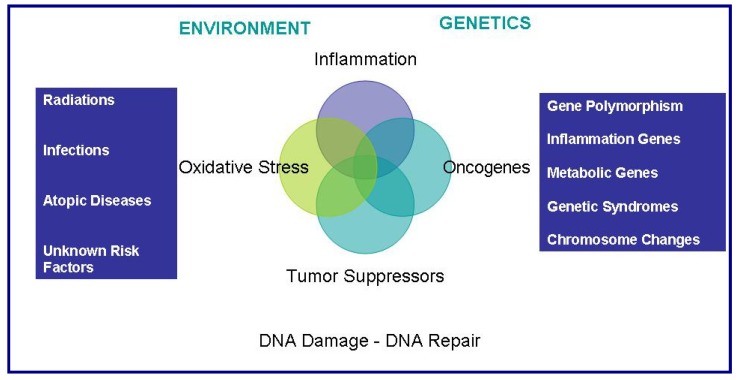
A possible interaction between environmental and genetic factors in gliomagenesis. Mediators of inflammation and oxidative stress pathways interacts with oncogenes and tumor suppressor genes influencing the evolution toward neoplastic phenotype by unbalancing the DNA repair/damage equilibrium.

Molecular epidemiology is a science that focuses on the contribution of potential genetic and environmental risk factors, identified at the molecular level, to the etiology, distribution and prevention of disease within families and across populations. Recently, genome-wide association studies with very large sample sizes and carefully matched controls have provided a powerful tool to identify genes involved in common human genetic diseases [76]. This emergent technology allowed the identification of susceptibility alleles providing a powerful instrument to understand mechanisms of carcinogenesis. Molecular epidemiology studies on gliomas detected an inverse association between IL-4, IL-4R, IL-13, and glioblastoma [77,78,79,80]. Furthermore, a meta-analysis of 3,450 gliomas and 1,070 meningiomas from eight observation studies found a strong inverse relationship between history of asthma, eczema, hay fever, or allergy and brain tumor [81]. The consistency of these findings suggests a possible role in gliomagenesis for immunologic factors and inflammation, clearly warranting more investigation of immune function genes (Figure 6).

## 7. MicroRNAs as Emerging Regulatory Molecules in Inflammation and Cancer

MicroRNAs (miRNAs) are a recently discovered class of small, evolutionary conserved RNA molecules that negatively regulate gene expression at the post-transcriptional level. The discovery of those small noncoding transcripts has broadened our understanding of the mechanisms that regulate gene expression with the addition of an entirely novel level of regulatory control. miRNAs consist of 18 to 25 nucleotides and represent a class of endogenous ribo-regulators that modulate gene expression via the RNA interference (RNAi) pathway. RNAi is a post-transcriptional silencing mechanism, present in most eukaryotic organisms, in which exposure to double-stranded RNA induces the sequence specific degradation of homologous messenger RNAs (mRNA). miRNAs act by base-pairing with their target mRNAs through perfect or nearly perfect complementarity, particularly at the 3′ untranslated regions (UTRs) of the target mRNAs [82,83,84,85,86] leading to their translational repression and/or direct cleavage [87]. 

MicroRNAs may play a role in the tumorigenesis and progression of cancer. Calin *et al.* first established a connection between microRNAs and cancer by showing that the miRNAs miR-15 and miR-16 are located at chromosome 13q14, a region deleted in more than half of B-cell chronic lymphocytic leukemia (CLL) [88]. Cimmino *et al.* then demonstrated that miR-15a and miR-16-1 expression was inversely correlated to Bcl-2 expression in CLL and that both miRNAs negatively regulated Bcl-2 at a posttranscriptional level. Furthermore, the Bcl-2 repression by these miRNAs induced apoptosis in a leukemic cell line model [89]. 

A role of miRNA has also been supposed in glioblastoma (GBM) the most common tumor of the brain and one of the most aggressive tumor in humans [90]. We recently demonstrated that miRNA 21 and 221 are up-regulated in human astrocytic tumors, whereas miRNA 181b is down-regulated. miRNA-21 was homogeneously overexpressed in low and high grade tumors, whereas miRNA-221 overexpression was more evident in high grade tumors [91].

Increased levels of miR-21 have been also found in several chronic inflammatory diseases [92]. The elevated levels of miR-21 in these tissues may be in part responsible for inflammation-associated cancers. Increased levels of miR-21 are found in nearly every malignancy examined and this increase is thought to be oncogenic [93]. miR-21 targets a number of tumor suppressor genes, including programmed cell death 4 (PDCD4) [94], tropomyosin 1 [95], phosphatase and tensin homolog (PTEN) [96], and BTG family member 2 [97]. Increased miR-21 expression can increase cell proliferation and inhibit apoptosis, whereas the inhibition of miR-21 can cause tumor regression in xenograft models [98]. Inflammatory stimuli can increase the expression of miR-21. It has been shown that IL-6 can induce the expression of miR-21 in a STAT3-dependent manner [99]. The EGFR pathway has also been shown to increase miR-21 expression [100]. Recently, miR-21 was found to directly target and repress IL-12-p35 expression in mouse models [101]. The miR-21- binding site in the 3’ UTR of IL12-p35 is conserved in humans. 

## 8. Conclusions

Much has been learned about the role of inflammation, inflammatory processes, and inflammation mediators in the pathogenesis of glioma. In fact, about one-third of the tumor burden is constituted by activated microglia/macrophages; in glioma cells genetic alterations affect the expression of various inflammatory genes and lead to recruitment of inflammatory cells. Overall, the tumor microenvironment is largely orchestrated by inflammatory mediators including cytokines, chemokines, reactive oxygen and nitrogen species, COX-2 and NF-κB that can create cellular conditions favorable for glioma promotion. Noticeably, in glioma the tumor microenvironment is an indispensable participant in the neoplastic process. Eventually, inflammatory molecules can cause reduction of cell-mediated cytotoxicity and potential immune evasion for tumors. All those biological characteristics, among others, make the glioma a “successful” tumor still carrying almost invariably an unfavorable prognosis. Further studies in this field, to identify possible therapeutic strategies, are warranted. 
